# Accelerated and standardized commissioning of a robotic RadioSurgery system using a hybrid golden beam data model

**DOI:** 10.1002/acm2.70705

**Published:** 2026-07-26

**Authors:** E. Pantelis, A. Moutsatsos, L. Sideri, P. Archontakis, A. Stergioula

**Affiliations:** ^1^ Radiotherapy Department Iatropolis Clinic Athens Greece; ^2^ Medical Physics Laboratory, School of Medicine National and Kapodistrian University of Athens Athens Greece

**Keywords:** commissioning, CyberComm, CyberKnife, golden beam data

## Abstract

**Background:**

The CyberKnife™ system requires extensive beam data acquisition across multiple collimation systems. The CyberComm™ framework integrates a linac output tuning procedure with a hybrid Golden Beam Data (GBD) model to accelerate and standardize CyberKnife commissioning while maintaining dosimetric accuracy.

**Purpose:**

In this study, CyberComm was employed for the commissioning of a CyberKnife model S7 system and evaluated with regard to its efficiency and dosimetric accuracy.

**Methods:**

Following the CyberComm framework, commissioning was separated into two stages. First, the linac beam was tuned by acquiring percentage depth dose (PDD) and off‐center ratio (OCR) measurements for the 60 mm fixed collimator (Cone‐60) and the primary (uncollimated) beam. These measurements were compared with corresponding reference datasets using one‐dimensional gamma index (GI) acceptance criteria of 1% (local)/1 mm for PDD, and 0.7% (global)/1 mm for OCR profiles. The process was repeated until GI passing rates exceeding 95% for all measurements. In the second stage, full beam commissioning measurements were performed for the smaller field sizes, while for the larger ones, the corresponding GBD values were validated through spot‐checks. Dosimetry measurements were conducted using a PTW‐60019 microdiamond detector. Dosimetric accuracy of the commissioned beam models was further evaluated using single beam and patient‐specific quality assurance (PSQA) measurements with the SRS MapCHECK™ detector.

**Results:**

Using vendor‐provided linac pre‐sets, the initial Cone‐60 PDD yielded a GI passing rate of 61%, while OCR profiles achieved 100%. After tuning the PFN‐HVPS voltage and steering coil currents, Cone‐60 PDD GI passing rate improved to 98%, with both primary beam and collimated OCR profiles achieving 100%. Spot‐check validation demonstrated excellent agreement between measured beam data and the GBD model, with 100% GI passing rates for TPR and OCR profiles across all evaluated field sizes. Output factors agreed within 0.4% with vendor reference data for the majority of fields, with larger deviations observed only for the smallest apertures. Single beam and PSQA irradiations showed increased dosimetric accuracy, with GI passing rates of 100% and ≥ 97%, respectively, using 2%/1 mm GI acceptance criteria. Complete commissioning of all collimation systems was achieved within 10 days.

**Conclusions:**

CyberComm‐assisted commissioning demonstrated accurate beam model generation for the CyberKnife system with a reduction in commissioning time relative to conventional protocols. Although these results are based on a single‐center installation, they support the clinical feasibility of this workflow for CyberKnife commissioning.

## 1 INTRODUCTION

In stereotactic radiosurgery (SRS), an ablative radiation dose is delivered to a well‐defined target in a single or a limited number of fractions.^1^, ^2^ Such doses can safely be delivered using radiotherapy treatment systems that provide sub‐millimeter target localization and generate dose distributions characterized by steep spatial dose gradients. Accurate target localization relies on detailed delineation of target contour upon high‐resolution anatomical images and precise determination of target position within the treatment room.^1^, ^2^, ^3^ Contouring of target and organs at risk (OARs) is performed on high resolution computed tomography (CT) and/or magnetic resonance imaging (MRI) image series. Steep spatial dose gradients are achieved using multiple non‐coplanar small radiation fields that collectively conform the dose distribution to the target, while sparing surrounding OARs. Beam shaping is commonly accomplished using cylindrical collimators or multi‐leaf collimators (MLCs) designed to minimize radiation field penumbra.^1^, ^2^, ^3^

The use of small fields in SRS introduces significant challenges in the determination of their dosimetric parameters during system commissioning. These challenges primarily arise from the loss of lateral charged particle equilibrium on the beam axis, partial occlusion of the primary photon source by the collimating devices, and the fact that detector dimensions may be comparable to or larger than the cross‐sectional beam dimensions at the depth of measurement.^4^, ^5^, ^6^, ^7^ These issues are addressed in corresponding small field dosimetry protocols, such as the IAEA TRS‐483 Code of Practice (CoP)^6^ and the American Association of Physicists in Medicine (AAPM) TG‐155 report,^7^ where guidance and detector‐specific data are provided to minimize systematic uncertainties. The AAPM estimates that commissioning a radiotherapy system may require several weeks, impacting equipment availability and departmental clinical workflow.^8^ The use of golden beam data (GBD) has been proposed as an alternative to conventional measurement‐based commissioning.^9^, ^10^, ^11^, ^12^ Successful implementation of this approach requires a beam‐matching procedure and high levels of mechanical and dosimetric reproducibility to ensure consistency between reference source models and individual treatment units.^13^, ^14^

The CyberKnife™ (CK) robotic SRS system (Accuray Inc., Madison, WI, USA) employs a compact linear accelerator (linac) mounted on a robotic manipulator.^15^, ^16^ The system supports multiple collimation systems and treatment planning dose calculation algorithms for small field radiation delivery. Commissioning of the required beam data typically spans from four to six weeks, depending on the available equipment, the number of personnel involved, and their level of experience. The recently released CyberComm™ framework (Accuray Inc) integrates linac tuning with a hybrid GBD model aiming to accelerate CK beam data commissioning and promote standardized outcomes across the user community. In this study the commissioning of a CK system using the CyberComm framework is reported and its dosimetric accuracy and commissioning efficiency is evaluated.

## 2 MATERIALS AND METHODS

### 2.1 The linear accelerator and beam collimation systems

The CK system employs a 6 MV flattening filter free (FFF) X‐ray linac operating at a nominal output rate of 800 or 1000 MU/min.^15^, ^16^ Radiofrequency (RF) power is generated by a 9.3 GHz (X‐band) magnetron. Using a waveguide, RF is delivered to a side‐coupled standing‐wave accelerating structure consisting of 37 cavities. Electrons are accelerated to a nominal kinetic energy of approximately 7 MeV prior to impinging on a tungsten target to produce bremsstrahlung X‐rays. Beam confinement is facilitated by two sets of tuning coils positioned along the accelerating structure; one near its midpoint and one near the electron gun. The produced X‐ray beam passes sequentially through a primary collimator, a monitor chamber, and an intermediate collimator designed to further reduce leakage radiation before reaching the secondary collimation system.^15^, ^16^

Three types of secondary collimation systems are available: A set of fixed cylindrical collimators (cones), a variable aperture collimator (Iris™), and a multi‐leaf collimator (MLC) (InCise™) for irregular field shaping.^15^, ^16^, ^17^, ^18^ There are twelve fixed cones available, producing circular radiation fields with nominal diameters ranging from 5 to 60 mm at a source‐to‐axis distance (SAD) of 800 mm. The Iris collimator consists of two opposing banks, each containing six independently movable diaphragm segments that adjust to form dodecagonal fields equivalent in size to the corresponding fixed cone diameters. The InCise MLC features 52 leaves arranged in two banks of 26, with a projected width of 3.85 mm at 800 mm SAD. This design allows the formation of radiation beams with nominal dimensions ranging from 7.7 × 7.6 mm^2^ to 115.0 × 100.1 mm^2^ at 800 mm SAD. To minimize penumbra and leakage radiation, the leaves are source‐focused, tilted 0.5° relative to the beam axis, and feature end geometries that remain source‐focused in all leaf positions (fully open, fully closed, and central) within the X‐ray field.

### 2.2 Beam data requirements for CK commissioning

The commissioning of a CK system necessitates a comprehensive suite of beam data including Tissue Phantom Ratios (TPRs), Off‐Center Ratios (OCRs), and Output Factors (OFs). These parameters must be determined across all nominal field sizes for the fixed, Iris and InCise MLC collimator systems.^19^ Table 1 summarizes the required beam data for each collimator system. Briefly, TPR data are required for a depth range spanning from 0 to 300 mm with 1 mm spatial resolution. OCR profile data are specified at minimum depths of 15, 100, and 300 mm. For collimators with nominal field diameters ≤ 20 mm, a spatial resolution of 0.2 mm or finer is required. For larger aperture sizes, the resolution may be relaxed to 0.5 mm. While orthogonal IEC‐X (crossline) and IEC‐Y (inline) OCR profiles are sufficient for the fixed and the InCise fields, profiles for the Iris collimator are collected at 0° (IEC‐X), 15°, 90° (IEC‐Y), and 105°. This angular sampling accounts for the dodecagonal aperture geometry of the Iris collimator and ensures characterization of both the flat edges and the vertices of the formatted fields.^17^ All OFs are normalized to the reference field created by the 60 mm fixed cone at 800 mm SAD. While InCise MLC OFs are defined at 800 mm SAD, additional OF data are required for the fixed and Iris collimators at 650 and 1000 mm SADs to ensure accurate characterization of the beam output across the clinical SAD range. For InCise MLC fields, the IEC‐X axis coincides with the MLC bank 1 to bank 2 direction, while the IEC‐Y axis is orthogonal to the leaf travel direction.

**TABLE 1:** Commissioning data requirements for the three CK secondary collimation systems.

Collimator type	Diameter^*^/field size^**^ (mm)	Dosimetric parameter (Depth(s) or SAD(s))	Measurement‐specific requirements
Fixed & Iris	5, 7.5, 10, 12.5, 15, 20, 25, 30, 35, 40, 50, 60	TPR (0 ‐ 300 mm), OCR (15, 100, 300 mm), OF (650, 800, 1000 mm)	fixed: IEC‐X, IEC‐Y profiles Iris: profiles at 0° (IEC‐X) 15°, 90° (IEC‐Y) and 105°
InCise MLC	(7.6, 7.7), (15.4, 15.4), (23.0, 23.1), (30.8, 30.8), (38.4, 38.5), (46.2, 46.2), (53.8, 53.9), (69.2, 69.3), (84.6, 84.7), (100.0, 100.1), (115.0, 100.1)	TPR (0 ‐ 300 mm), OCR (15, 100, 300 mm), OF (800 mm)	IEC‐X, IEC‐Y profiles

* Nominal diameters for the fixed and Iris collimators are defined at an 800 mm SAD.

** InCise MLC field sizes are specified as nominal (IEC‐X, IEC‐Y) dimensions at an 800 mm SAD.

Dose calculations within the Precision™ Treatment Planning System (TPS) (Accuray Inc.) are performed using one of the three available algorithms: the *Ray‐Tracing* (RT) algorithm which is assigned to fixed and Iris collimators; the *Finite Size Pencil Beam (FSPB)* algorithm appointed to the InCise MLC; and the *Monte Carlo (MC)* algorithm, which is available for all secondary collimation systems.^20^ For *Ray‐Tracing*, beam modeling essentially comprises a direct mapping of the commissioned TPR and OCR data into a high‐resolution lookup table for rapid dose reconstruction. Commissioning the *MC* and *FSPB* algorithms necessitates specific supplemental data. These include: a) Percent Depth Dose (PDD) data measured at an 800 mm Source‐to‐Surface Distance (SSD) for both the fixed and Iris collimator fields of 60 mm nominal diameter, b) open‐field IEC‐X and IEC‐Y OCR profiles at 25 mm depth and 800 mm SAD, acquired with the secondary fixed collimator housing in place but without a cone loaded, and c) IEC‐X, IEC‐Y, and diagonal (45° and 135°) OCR profiles of the primary beam, sampled at a depth of 20 and 800 mm SAD without any collimator housing loaded.

### 2.3 The CyberComm framework

**FIGURE 1:**
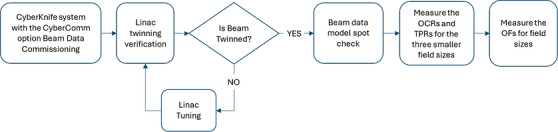
The CyberComm workflow for CK beam data commissioning.

The CyberComm framework combines a vendor‐defined reference beam dataset and a hybrid GBD model, determined from the average of ten matched CK linacs. Unlike traditional commissioning, which relies entirely on site‐specific measurements, this framework follows a structured, two‐stage workflow (Figure 1). The first stage involves beam matching, where the linac's beam characteristics are tuned to align with the reference dataset. The second stage consists of GBD model validation (spot checks) across representative field sizes and secondary collimator systems. Comparison of the acquired beam data with the corresponding reference values for both stages was performed using the BeamCheck v.1.0 dedicated software tool provided by the vendor.

#### 2.3.1 Linac tuning

**FIGURE 2:**
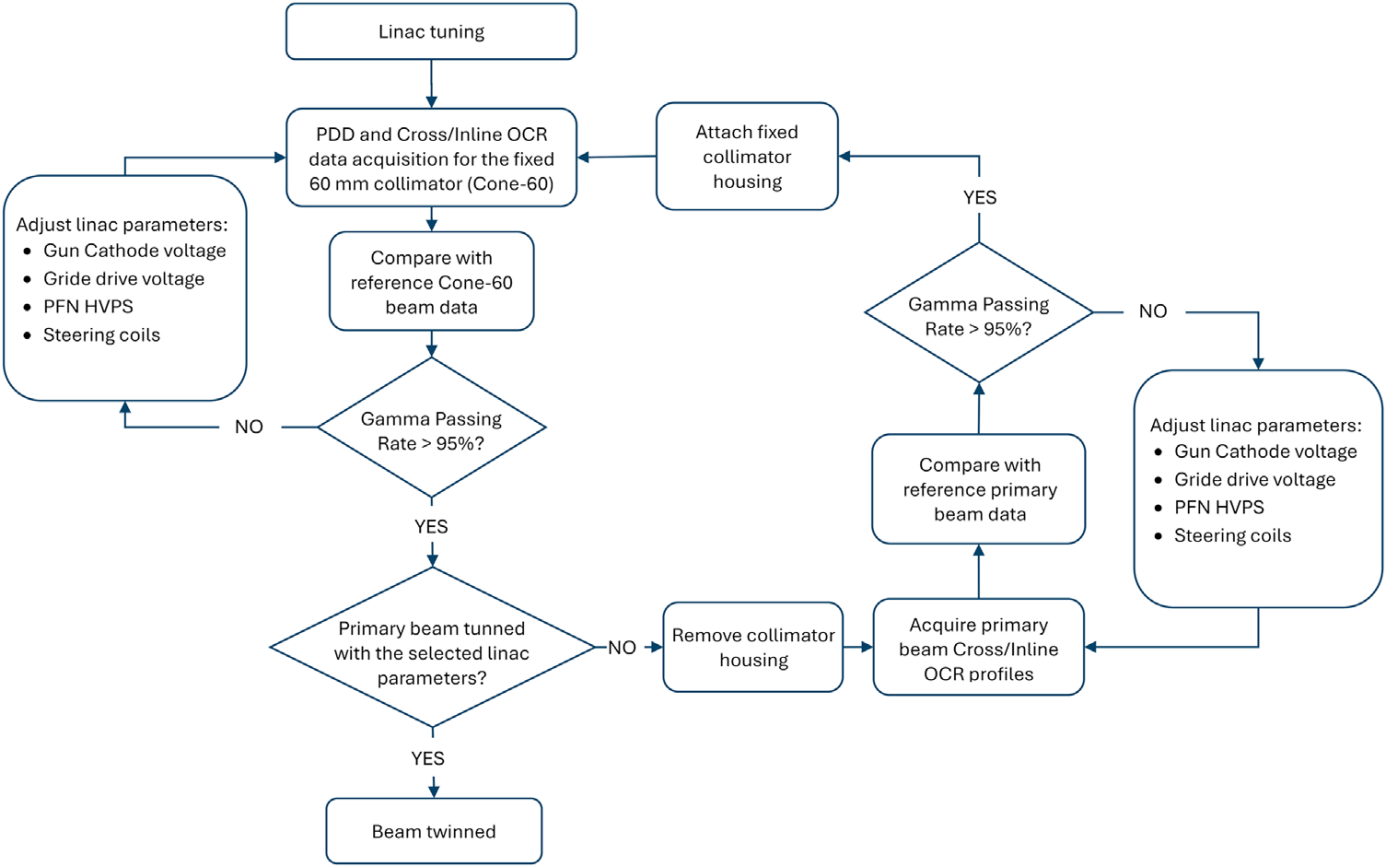
The CyberComm workflow for matching the linac beam characteristics to the reference dataset.

The workflow for matching the linac beam characteristics with the reference dataset is presented in Figure 2. PDD and orthogonal (IEC‐X/IEC‐Y) OCR profiles are acquired for the 60 mm fixed cone at an SSD of 800 mm. PDDs are measured from 0 to 300 mm, while OCR profiles are measured from ‐40 to 40 mm at a depth of 50 mm. These measurements are compared against the corresponding reference dataset to evaluate beam‐match quality. Matching is confirmed using 1D gamma index (GI) analysis, utilizing vendor‐recommended criteria of 1% (local)/ 1 mm for the PDD and 0.7% (global)/1 mm for the OCR profiles. A GI passing rate >95% is required for a successful match. If this threshold is met, the primary beam is verified by acquiring IEC‐X and IEC‐Y OCR profiles at a 50 mm depth, over a range of ‐80 to 80 mm. These profiles are subsequently compared against the reference dataset using the same 0.7%/1 mm global GI criteria. If primary beam profiles also yield a GI passing rate >95%, the linac is considered matched and beam data commissioning proceeds. If the 60 mm fixed cone or primary beam data do not match the reference dataset, the following linac parameters are adjusted: (a) gun cathode voltage, (b) grid drive voltage, (c) pulse forming network—high voltage power supply (PFN‐HVPS), and (d) the currents of the two sets of beam steering coils (X1/Y1 and X2/Y2). Among these, the PFN‐HVPS and grid drive voltage primarily influence beam energy. Increasing the PFN‐HVPS raises beam energy, which subsequently increases the PDD. On the other hand, increasing the grid drive increases the beam current, but for the same input power, the average energy transferred to the electrons is lower due to increased beam‐loading. The grid drive and grid cathode affect the dimensions of the focal spot and consequently the shape of the IEC‐X and IEC‐Y OCR profiles. The first set of steering coils (X1/Y1) controls the position of the focal spot along the IEC‐Y and IEC‐X axes, while the second set (X2/Y2) adjusts the beam tilt.

#### 2.3.2 Spot check

Following linac matching, a systematic spot check procedure is performed to verify the dosimetric coincidence between the local CK system and the GBD model. The validation protocol is stratified by collimator system and field size (Table 2). For the fixed and Iris collimators, TPR validation is limited to the 12.5 and 60 mm nominal diameter fields, whereas OCR profiles are verified at a 100 mm depth for all available fields with nominal diameter ≥ 12.5 mm. For the InCise MLC, validation is performed at minimum for the 30.8 × 30.8 mm^2^ and 115.0 × 100.1 mm^2^ fields, with OCRs acquired at 100 mm depth. To mitigate small field uncertainties, the TPR and OCR data for the fixed and Iris collimator fields with nominal diameters of 5 mm, 7.5 and 10 mm are directly measured and merged with the GBD model. Similarly, the MLC fields with dimensions of 7.6 × 7.7 mm^2^, 15.4 × 15.4 mm^2^ and 23.0 × 23.1 mm^2^ are individually characterized and integrated. Finally, OFs are measured for every commissioned beam and collimator system (Table 2).

**TABLE 2:** Summary of commissioning workflow requirements for the three secondary CK collimation systems under the CyberComm framework. Measurements, golden beam data and validation spot checks of the latter are denoted with *m*, GBD and GBD‐SC, respectively.

		Field Size (mm)											
Fixed & Iris		5	7.5	10	12.5	15	20	25	30	35	40	50	60
OCR depth (mm)	15	m	m	m	GBD	GBD	GBD	GBD	GBD	GBD	GBD	GBD	GBD
	100	m	m	m	GBD‐SC	GBD‐SC	GBD‐SC	GBD‐SC	GBD‐SC	GBD‐SC	GBD‐SC	GBD‐SC	GBD‐SC
	300	m	m	m	GBD	GBD	GBD	GBD	GBD	GBD	GBD	GBD	GBD
TPR	‐	m	m	m	GBD‐SC	GBD	GBD	GBD	GBD	GBD	GBD	GBD	GBD‐SC
OF SAD (mm)	650	m	m	m	m	m	m	m	m	m	m	m	m
	800	m	m	m	m	m	m	m	m	m	m	m	m
	1000	m	m	m	m	m	m	m	m	m	m	m	m

MLC		7.6 × 7.7	15.4 × 15.4	23.0 × 23.1	30.8 × 38.5	46.2 × 46.2	53.8 × 53.9	69.2 × 69.2	84.6 × 84.7	100 × 100.1	115 × 100.1		
OCR depth (mm)	15	m	m	m	GBD	GBD	GBD	GBD	GBD	GBD	GBD		
	100	m	m	m	GBD‐SC	GBD	GBD	GBD	GBD	GBD	GBD‐SC		
	300	m	m	m	GBD	GBD	GBD	GBD	GBD	GBD	GBD		
TPR	‐	m	m	m	GBD‐SC	GBD	GBD	GBD	GBD	GBD	GBD‐SC		
OF SAD (mm)	800	m	m	m	m	m	m	m	m	m	m		

Prior to beam data commissioning, additional calibration procedures are performed for the Iris and InCise MLC systems to ensure that the generated field sizes align with those defined in the GBD model. Specifically, a garden fence test is performed to calibrate the InCise MLC leaf positions.^21^ The calibration of the Iris collimator is based on water phantom measurements where the generated field sizes—defined by the Full Width at Half Maximum of OCR profiles acquired at 100 mm depth and 800 mm SAD—are adjusted to agree within ±0.2 mm to the GBD reference values.

Quantitative agreement is assessed using 1D GI analysis with a target passing rate of >95% for beam data acceptance. Specifically, gamma criteria of 1.5% (local)/1 mm are used for TPR validation. For OCR profiles, distance‐to‐agreement (DTA) criteria of 0.3‐1.0 mm and global dose difference (DD) criteria of 0.5%‐1.5% are used, scaled according to field size and measurement depth. Any profile exceeding these spot check tolerances necessitates comprehensive direct measurement.

### 2.4 Linac tuning and beam data measurements

All dosimetric measurements were performed using an MP3 motorized water phantom (PTW Freiburg GmbH, Freiburg, Germany). The phantom was aligned such that the vertical scanning axis coincided with the beam central axis within 0.2 mm over a depth range of 50‐250 mm, while the in‐plane scanning axes were aligned to coincide with the IEC‐X and IEC‐Y axes. Linac matching measurements were performed using a PTW‐31021 Semiflex 3D ionization chamber mounted with its stem parallel to beam central axis in the water phantom. In this orientation, the effective point of measurement is located 1.7 mm below ionization chamber's physical external tip. Measurements were acquired in step mode, with a step resolution of 0.5 mm, a measurement time of 0.2 s per step, and a scanning speed of 2 mm/s.^22^

Beam data measurements were performed using a PTW‐60019 synthetic microdiamond detector aligned in axial orientation in the water phantom.^23^, ^24^ A PTW‐31010 Semiflex ionization chamber was used as a reference detector to correct for output fluctuations during relative dose measurements. It should be noted that both the PTW‐31021 and ‐60019 detectors were used by the vendor to generate the reference dataset for linac matching and for the development of the GBD model, respectively. TPR measurements were performed in continuous mode with 1 mm spatial resolution over a depth range of 0 ‐ 300 mm (Table 1). OCR profiles were acquired in step mode with a measurement time of 0.2 s per step and a scanning speed of 2 mm/s. OCR range and step size were tailored to the collimator type and field size. For the fixed and Iris collimators, a range of ± 30 mm with a step of 0.2 mm was used for nominal field diameters ≤ 20 mm, and ± 50 mm with a step of 0.5 mm for larger fields. For the MLC collimator, a range of ± 80 mm was used for all fields except the 115 × 100.1 mm^2^ field, where the range was extended to ± 125 mm (Table 2). For the MLC fields with dimensions ≤ 30.8 × 30.8 mm^2^ a measurement step of 0.2 mm was used for off center distances up to 30 mm, increasing to 5 mm beyond. For the 115 × 100.1 mm^2^ field, a measurement step of 0.5 mm was used up to 70 mm, also increasing to 5 mm beyond.

The OFs, $\Omega_{Q_{clin},\;Q_{msr}}^{f_{clin},f_{msr}}$, were determined according to:

$$\;\Omega_{Q_{clin},\;Q_{msr}}^{f_{clin},f_{msr}}=\frac{M_{Q_{clin}}^{f_{clin}}}{M_{Q_{msr}}^{f_{msr}}}*K_{Q_{clin},\;Q_{msr}}^{f_{clin},f_{msr}},$$

where the $M_{Q_{clin}}^{f_{clin}}$ and $M_{Q_{msr}}^{f_{msr}}$ represent the measured charge for the clinical fields (*f_clin_*) and the 60 mm fixed cone machine‐specific reference field (*f_msr_*), respectively. All measurements were performed at the reference depth of 15 mm. The $K_{Q_{clin},\;Q_{msr}}^{f_{clin},f_{msr}}$ term represents the field size dependent correction factor for the PTW‐60019 microdiamond detector, accounting for the beam conditions specific to CK small field dosimetry.^25^, ^26^, ^27^, ^28^ It is noted that the ratio $M_{Q_{clin}}^{f_{clin}}/M_{Q_{msr}}^{f_{msr}}$ was also determined representing the measured OFs, for reasons of comparison with the corresponding vendor‐provided reference dataset. Detector positioning was verified by acquiring IEC‐X and IEC‐Y profiles with 0.1 mm step size for the smallest field size available with each collimator system. A minimum of five independent measurements were performed for each field size, with 100 MU delivered per irradiation to ensure a stable signal‐to‐noise ratio. To address the mechanical reproducibility of the Iris collimator, the aperture was fully opened and re‐established between each irradiation.^29^ Measured data for each field size were averaged, and the measurement uncertainty was estimated as the standard deviation of the mean.

### 2.5 Dosimetric verification

The dosimetric accuracy of the CyberComm framework was evaluated using single‐beam plans and pre‐treatment patient‐specific quality assurance (PSQA) irradiations. For the PSQA measurements, the clinical information of the cases evaluated, and details of the corresponding treatment plans are summarized in Table 3. Besides technical information, the Paddick conformity index (CI)^30^ and the gradient index (GI)^31^ of the delivered dose distributions are also tabulated as plan quality descriptors. Additionally, a complexity descriptor is presented for each plan in terms of plan irregularity (PI), calculated along the lines described in Du *et al.* ^32^ It is noted that PI is, by definition, equal to 1 for treatment plans irradiated with the circular fields formed by the fixed and Iris collimators. The higher the PI value the greater the deviation of the irradiated fields from the circular‐shape.

**TABLE 3:** Summary of PSQA plan and patient details.

				Aperture (mm)					
A/A	Anatomical Site	Target Volume (cm^3^)	Collimator system	min	max	No of Beams / Segments	Dose Calculation Algorithm^*^	CI/GI	PI
1	Head	2.91	fixed	7.5	12.5	108	RT (CC)	0.87/3.58	1.00
2	Head	4.33	fixed	7.5	15	160	RT (CC)	0.96/2.75	1.00
3	Head	10.77	fixed	7.5	12.5	170	RT (CC)	0.93/2.83	1.00
4	Head	10.65	fixed	12.5	20	160	RT (CC)	0.92/2.97	1.00
5	Head	11.94	Iris	70.5	12.5	139	RT (CC)	0.93/2.51	1.00
6	Lung	15.63	Iris	20	30	58	RT (CC)	0.89/3.78	1.00
7	Head	4.23	Iris	7.5	12.5	98	RT (CC)	0.89/2.65	1.00
8	Lung	5.14	Iris	30	30	20	RT (CC)	0.82/6.29	1.00
9	Liver	27.29	Iris	15	35	145	RT (CC)	0.81/6.21	1.00
10	Lung	11.05	InCise MLC	10.1	32	28/30	FSPB (LS)	0.83/4.09	1.63
11	Lung	6.21	InCise MLC	10.3	26.5	35/39	FSPB (LS)	0.73/4.78	1.80
12	Lung	5.60	InCise MLC	8.5	23.4	31/44	FSPB (LS)	0.86/5.01	1.60
13	Lung	8.89	InCise MLC	15	27.1	24/30	FSPB (LS)	0.81/4.02	1.54
14	Lung	92.66	InCise MLC	7.8	48.7	32/39	FSPB (LS)	0.85/2.98	2.13
15	Adrenal	33.20	InCise MLC	8.5	37.3	34/40	FSPB (LS)	0.89/3.46	1.80

* RT: Ray Tracing with Contour Correction enabled; FSPB (LS): FSPB with Lateral Scaling correction enabled; CI: Paddick Conformity Index; GI: Gradient Index; PI: Plan Irregularity.

Dosimetry measurements were performed using the SRS MapCHECK (Sun Nuclear Inc., Melbourne, FL, USA) diode array detector. The SRS MapCHECK features 1,013 n‐type diodes, each with an active area of 0.48 × 0.48 mm^2^ and a sensitive volume of 0.007 mm^3^, arranged with a spatial resolution of 2.47 mm. Four gold fiducials are also embedded in the periphery of the detector to facilitate image‐guided CK fiducial tracking. For dose measurements, the detector was enclosed within two PMMA slabs and inserted into the StereoPHAN™ phantom (Sun Nuclear Inc.). The StereoPHAN phantom assembly, containing the SRS MapCHECK, was imaged using an Aquilion™ LB Computed Tomography (CT) simulator (Canon Medical Systems Inc., Japan) following the acquisition protocol used for CK intracranial treatments: 120 kV, 400 mAs, 1 mm slice thickness, 280 cm field of view and 512 × 512 reconstruction matrix.^33^ The imaging data were imported into the Precision TPS, where three primary structures were contoured: the central diode detector, the MLC target, and the external body.

A total of 15 single beam and 15 clinical treatment plans were evaluated involving the fixed, Iris and MLC collimator systems and the RT and FSPB dosimetric algorithms (i.e., MC was not evaluated). For each single beam irradiation, the TPS was used to create a plan delivering a dose of 2 Gy at the central diode of the detector. For PSQA plan, the TPS was used to map the irradiation geometry, involving the employed collimator system and aperture sizes, linac positions and directions, and delivered MU per aperture size, onto the StereoPHAN phantom with the SRS MapCHECK detector in place. The dose distribution was aligned with the detector's plane in each case, and recalculated using the corresponding dose calculation algorithm. In all cases the dose calculation grid covered the entire CT volume with a voxel size of (0.55 × 0.55 × 1) mm^3^. A density override of 1.2 g/cm^3^ was applied to the PMMA components in accordance with the manufacturer's specifications. The calculated dose datasets, along with the “results.xml” file containing the plan data, was exported from the TPS and imported into the SNC Patient software (version 8.3). Measured dose data were compared with the TPS‐calculated dose distributions using GI analysis within the SNC Patient software. Additional correction factors, including angular, dose rate, field size, and temperature corrections, were applied within the SNC Patient software. Different GI acceptance criteria were applied. For single beam plans global DD of 2% and 1% were used with a 5% dose threshold.^34^ For PSQA plans, DD criteria ranging from 3% to 1% with a 10% dose threshold were used.^35^ In all cases a distance‐to‐agreement (DTA) criterion of 1 mm was used. Further details on the application of this system for CK dosimetry measurements are available in the literature.^35^

## 3 RESULTS

### 3.1 Linac tuning

**FIGURE 3:**
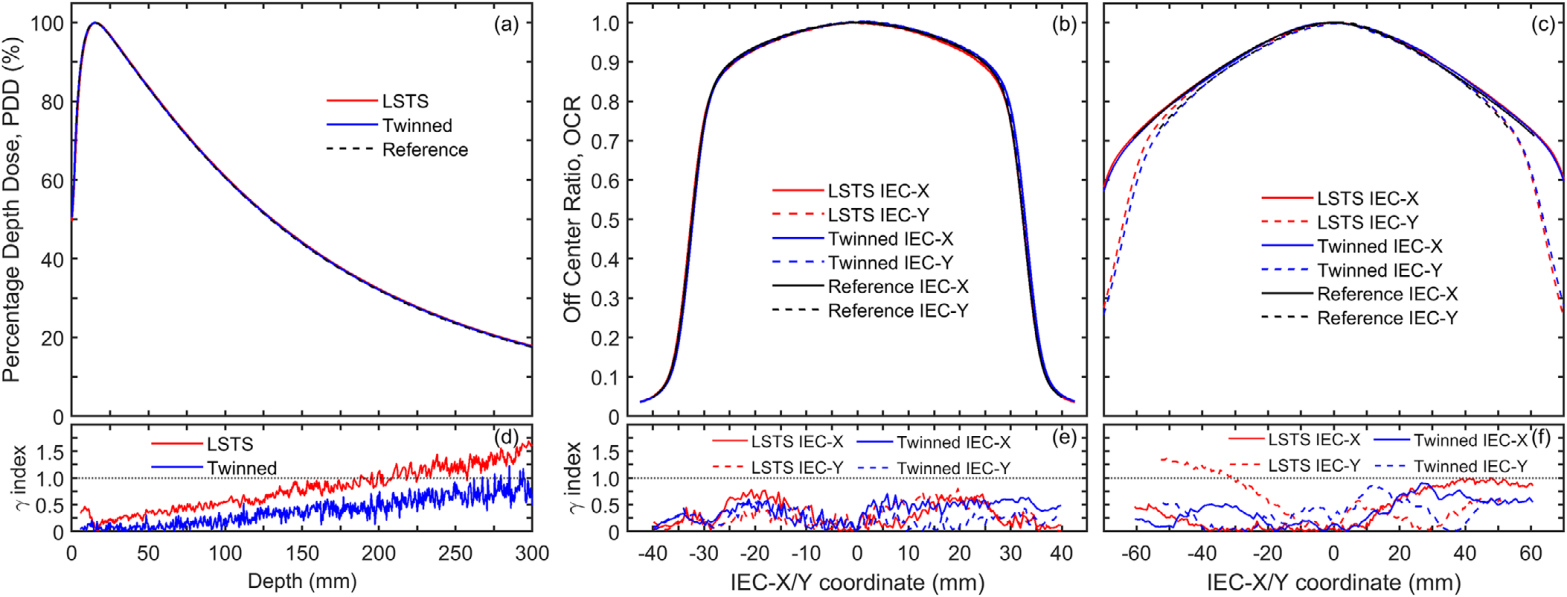
(a, b) PDD and OCR profiles for the 60 mm Fixed collimator (Cone‐60), and (c) primary beam OCR profiles, measured using LSTS settings (in red) and final tuned settings (in blue). Reference linac matching data are also shown (in black). One‐dimensional gamma (γ) index analysis is presented for (d) PDD profiles using 1% (local)/1 mm criteria and (e, f) OCR profiles using 0.7% (global)/1 mm criteria.

All CK linacs with the CyberComm option are factory‐tested and matched to the reference beam dataset, with operating parameters documented in the vendor‐supplied Linac Subsystem Test Summary (LSTS) report. Following installation, these LSTS parameters are implemented on‐site and used to verify linac matching. Using the LSTS settings, the 60 mm fixed cone PDD and OAR profiles are shown in Figure 3. As can be observed, the LSTS configuration achieved a 100% GI passing rate for both IEC‐X and IEC‐Y OCR profiles. However, a lower GI passing rate of 61% was observed for the PDD, indicating a beam energy mismatch (the energy of the installed linac was slightly higher than the reference data). In addition, primary beam validation revealed minor steering asymmetries that were less evident in the collimated field (Figure 3b, c). Specifically, while the IEC‐X primary beam profile showed high agreement (98% GI passing rate), the IEC‐Y profile exhibited a reduced GI passing rate of 78%.

To resolve these discrepancies, adjustments were made to the PFN‐HVPS and steering coil currents. The PFN‐HVPS was reduced from 14.91 kV to 14.51 kV to decrease the beam energy, and the current of the X1 steering coil was adjusted from 0.03 A to 0 A to re‐center the focal spot. Following these refinements, the GI passing rate for the 60 mm cone PDD improved to 98%, while both the primary beam and 60 mm cone profiles achieved 100% passing rates (Figure 3) and the linac was considered matched.

### 3.2 Spot check and beam data commissioning

**FIGURE 4:**
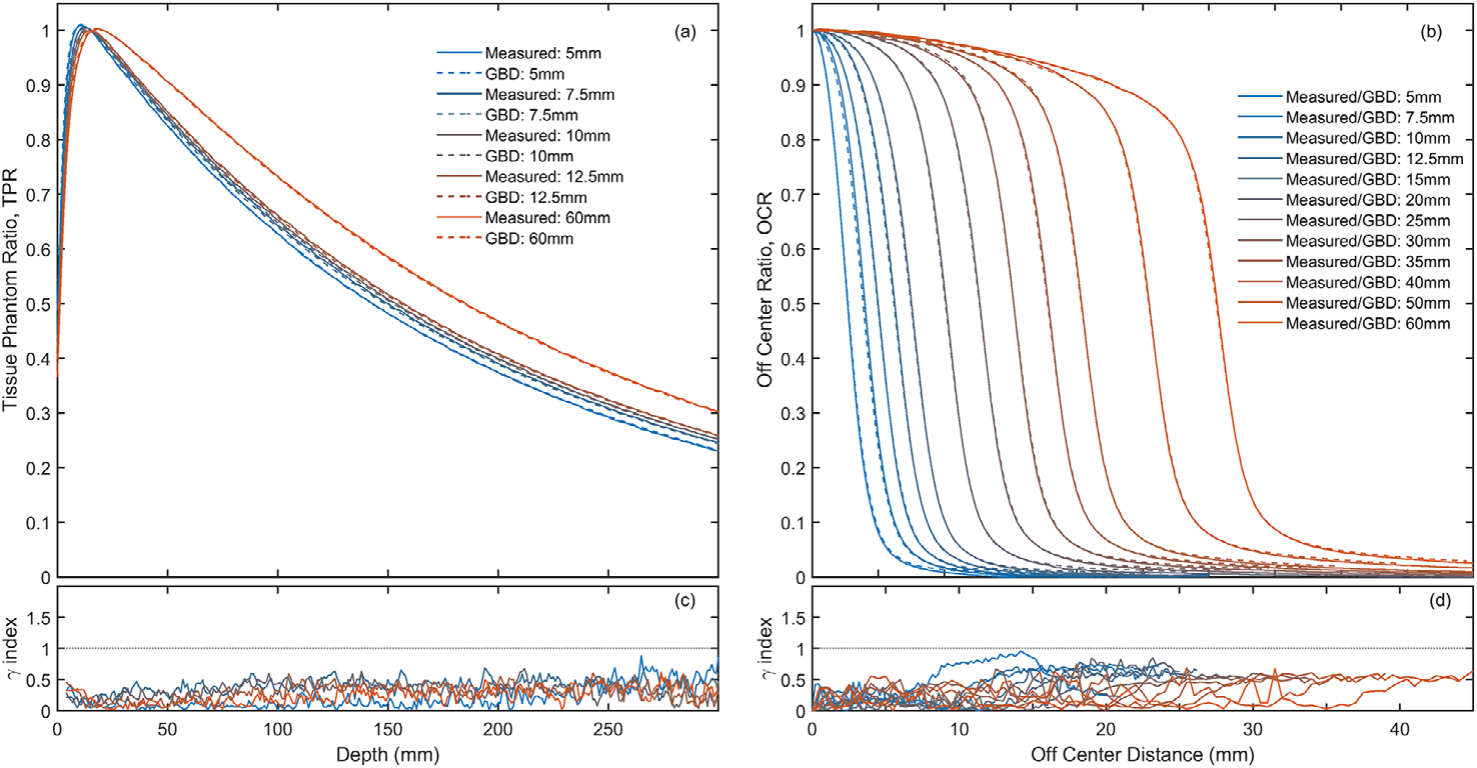
Comparison between measured (solid lines) and GBD model (dashed lines) data: (a) TPR and (b) OCR profiles for the fixed collimators. Corresponding 1D gamma (γ) index analysis results are provided for: (c) TPR profiles using 1.5% (local)/1 mm criteria; and (d) OCR profiles using 0.6% (global)/0.3 mm criteria for nominal field diameters ≤ 20 mm, and 0.6% (global)/0.6 mm criteria for field diameters > 20 mm.

**FIGURE 5:**
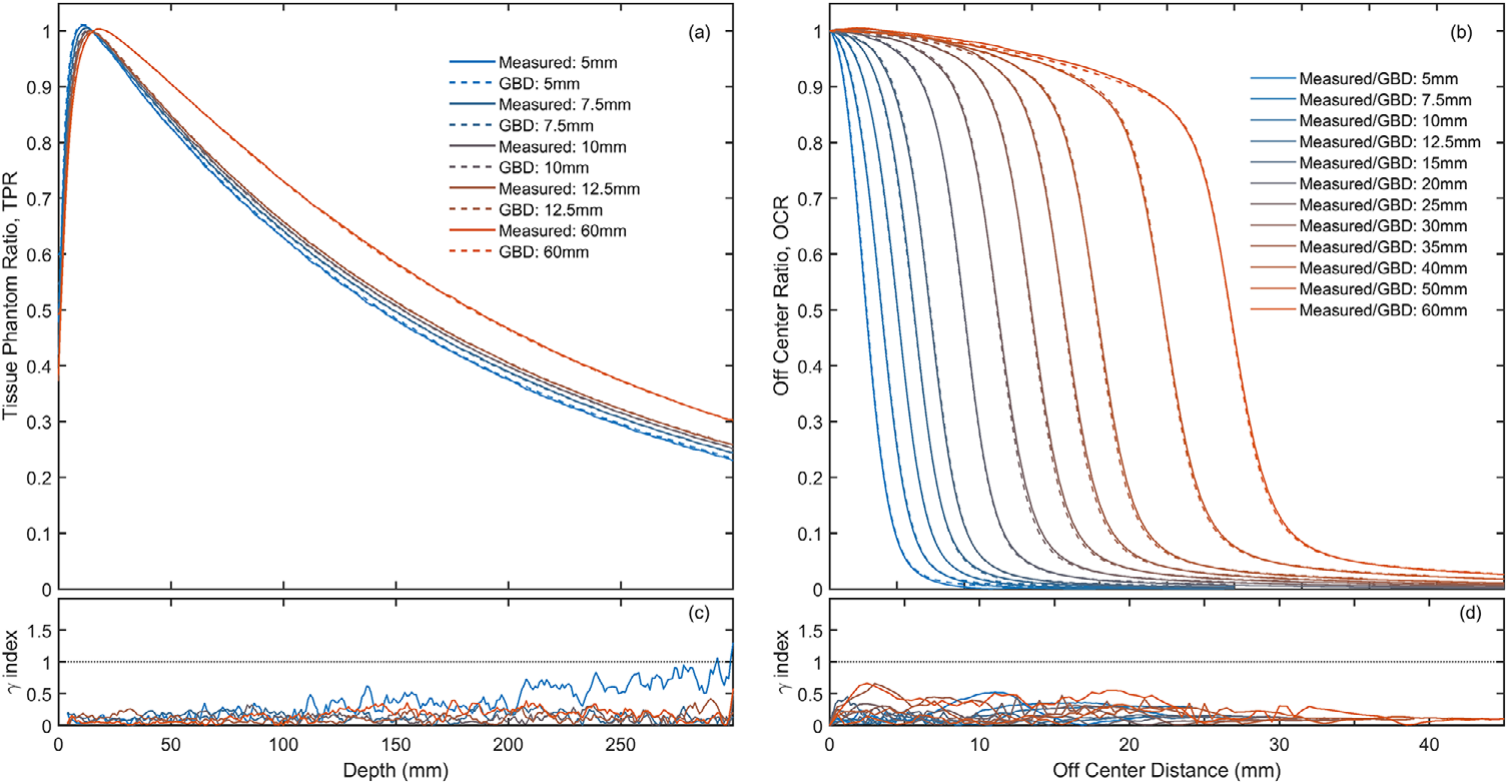
Comparison between measured (solid lines) and GBD model (dashed lines) data: (a) TPR and (b) OCR profiles for the Iris collimator. Corresponding 1D gamma (γ) index analysis results are provided for: (c) TPR profiles using 1.5% (local)/1 mm criteria; and (d) OCR profiles using 0.6% (global)/0.3 mm criteria for nominal field diameters ≤ 20 mm, and 0.6% (global)/0.6 mm criteria for field diameters > 20 mm.

**FIGURE 6:**
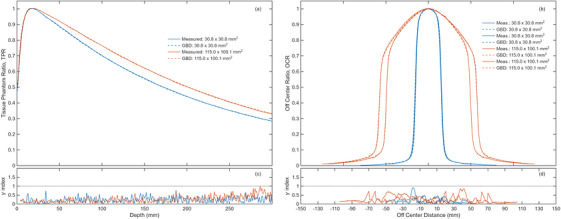
Comparison between measured (solid lines) and GBD model (dashed lines) data: (a) TPR and (b) OCR profiles for the InCise MLC 30.8 × 30.8 mm^2^ and 115.0 × 100.1 mm^2^ nominal field sizes at 800 mm SAD. Corresponding 1D gamma (γ) index analysis results are provided for: (c) TPR profiles using 1.5% (local)/1 mm criteria; and (d) OCR profiles using 1% (global)/0.5 mm criteria for the 30.8 × 30.8 mm^2^ and 1% (global)/ 1 mm criteria for the 115.0 × 100.1 mm^2^ field size.

In Figures 4, 5, 6, TPR and OCR verification measurements for the fixed cone, Iris, and MLC beam models are presented. Corresponding GBD data are also included for reasons of comparison. Presented data reveal precise depth‐dose matching and lateral beam consistency. In more detail, comparison of the presented TPR data for each collimator type demonstrated agreement within 1.5% over the entire evaluated depth range. Similarly, OCR comparisons showed agreement within 2% down to the 5% OCR level for the evaluated fields for each collimator type. One‐dimensional (1D) GI analysis yielded 100% passing rates for both TPR (Figures 4c, 5c, 6c) and OCR profiles (Figures 4d, 5d, 6d).

**FIGURE 7:**
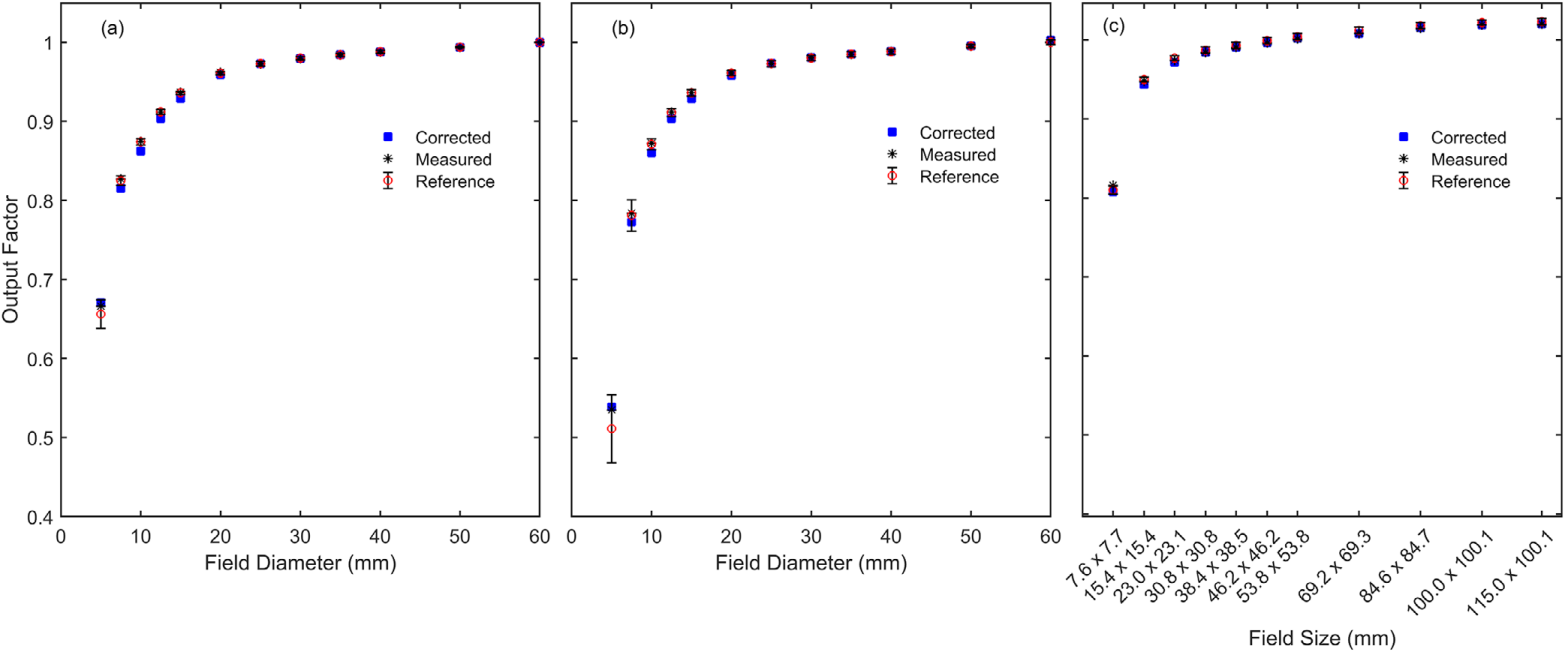
$\Omega_{Q_{\mathrm{clin}},\;Q_{\mathrm{msr}}}^{f_{\mathrm{clin}},f_{\mathrm{msr}}}$ data for the commissioned field sizes using the fixed (a), Iris (b), and InCise MLC (c) collimation systems at 800 mm SAD. Corresponding measured and vendor‐supplied reference OF data are also plotted for reasons of comparison. The reference OFs represent mean values acquired with PTW‐60019 detectors across ten systems without applying any small field corrections, with error bars indicating ± 2 standard deviations.

In Figure 7 the $\Omega_{Q_{clin},\;Q_{msr}}^{f_{clin},f_{msr}}$ data for the commissioned field sizes at 800 mm SAD are presented for the fixed, Iris and InCise MLC collimator systems. For reasons of comparison each plot includes the corresponding measured OFs and the reference dataset. It is noted that the reference OF dataset represents mean uncorrected OF values acquired with PTW‐60019 detectors across ten matched CK systems, with error bars indicating ± 2 standard deviations.

At 800 mm SAD, measured output factors (OF) for the fixed collimators agreed within 0.3% with the reference dataset for all field sizes, except for the 5 mm cone where a deviation of 1.5% was found. Similar trends were observed at 650 and 1000 mm SADs (data not shown); for the 5 mm cone, deviations were 1.0% and 2.1%, respectively. Regarding the Iris collimator, OF values at 800 mm SAD agreed within 0.5% with corresponding reference data for all field sizes except for the 5 mm cone, where a deviation of 4.6% was calculated. At the 650 and 1000 mm SADs (data not shown), agreement for the Iris was reached within 0.6% (650 mm) and 0.9% (1000 mm) for all field sizes except for the 5 mm field, for which larger discrepancies up to 4.4% and 4.8%, respectively, were found. Finally, the MLC collimator exhibited the closest agreement with the reference datasets within 0.8% across all measured field sizes.

### 3.3 CyberComm commissioning efficiency

The preconfigured linac enabled efficient beam tuning, requiring minimal iterative adjustments and approximately 3 hours to complete. Spot‐check and beam data commissioning for all secondary collimation systems were completed within 10 days. Assuming a typical beam data commissioning period of 25 days using conventional workflows, this time reduction corresponds to an efficiency gain of approximately 60%.

### 3.4 Single beam dose verification

Single‐beam dose verification results for the fixed, Iris and InCise MLC collimator systems are summarized in Table 4. An excellent dosimetric accuracy can be observed with 100% GI passing rates using 2%/1 mm acceptance criteria. Using the 1%/1 mm acceptance criteria, GI passing rates of 100% were achieved for all single beam plans except those using small‐field apertures, where passing rates decreased to ≥ 97%.

**TABLE 4:** GI passing rates for the single‐beam irradiations using the fixed, Iris and InCice MLC collimator systems and two of the available dose calculation algorithms. Gamma calculations were performed using for two different global GI acceptance criteria.

				GI passing rate (%)	
AA	Collimator type	Field size (mm)	Dose calculation algorithm	2%/1 mm	1%/1 mm
1	fixed	10	RT (CC)	100	97
2	fixed	15	RT (CC)	100	100
3	fixed	20	RT (CC)	100	100
4	fixed	25	RT (CC)	100	100
5	fixed	30	RT (CC)	100	100
6	Iris	10	RT (CC)	100	97
7	Iris	15	RT (CC)	100	100
8	Iris	20	RT (CC)	100	100
9	Iris	30	RT (CC)	100	100
10	Iris	40	RT (CC)	100	100
11	InCise MLC	15.4 × 15.4	FSPB (LS)	100	98
12	InCise MLC	23.0 × 23.1	FSPB (LS)	100	100
13	InCise MLC	30.8 × 30.8	FSPB (LS)	100	100
14	InCise MLC	38.4 × 38.4	FSPB (LS)	100	100
15	InCise MLC	53.8 × 53.8	FSPB (LS)	100	100

### 3.5 Patient specific dose verification

In Table 5, the PSQA verification results for the fixed, Iris, and InCise MLC collimators are summarized. Among the presented plans, six involved intracranial treatments treated using the fixed (four) or the Iris (two) collimators, while nine involved extracranial cases treated using the Iris (three) and the InCise MLC (six) collimators. A variety of field sizes were used ranging from 7.5 mm to 48.7 mm with the majority of treatment plans utilizing the fixed or the Iris collimators to involve two or more field sizes. Moreover, intracranial plans utilized higher number of beams, whereas extracranial cases utilized the InCise MLC resulting in a smaller number of beams. High dosimetric accuracy was in general observed, with all plans achieving GI passing rates ≥ 98% at the 3%/1 mm and ≥ 97% at the 2%/1 mm acceptance criteria. For the tighter 1%/1 mm GI acceptance criteria, passing rates decreased to ≥ 92%, primarily for plans using small‐field apertures. No trend related to the secondary collimator system was observed.

**TABLE 5:** GI passing rates for the PSQA irradiations using the fixed, Iris and InCice MLC collimator systems and two of the available dose calculation algorithms. Gamma calculations were performed for three different global GI acceptance criteria.

				GI passing rate (%)		
AA	Anatomical Site	Dose Calculation Algorithm	Fraction Dose (cGy)	3%/1 mm	2%/1 mm	1%/1 mm
1	Head	RT (CC)	800	100	100	97
2	Head	RT (CC)	2000	99	97	94
3	Head	RT (CC)	600	100	100	92
4	Head	RT (CC)	600	100	100	98
5	Head	RT (CC)	500	98	97	93
6	Lung	RT (CC)	1200	100	100	98
7	Head	RT (CC)	900	98	97	95
8	Lung	RT (CC)	1800	100	100	100
9	Liver	RT (CC)	1800	100	100	100
10	Lung	FSPB (LS)	1250	100	100	92
11	Lung	FSPB (LS)	1800	100	100	95
12	Lung	FSPB (LS)	1800	100	100	100
13	Lung	FSPB (LS)	1600	100	100	99
14	Lung	FSPB (LS)	1100	100	100	95
15	Adrenal	FSPB (LS)	700	100	99	93

## 4 DISCUSSION

In this study, the efficiency and dosimetric accuracy associated with the use of the CyberComm framework for the commissioning of the CK Precision TPS were evaluated. Linac tuning procedure was found effective, with small adjustments in beam energy and focal spot position required to achieve matching with the reference beam data. The spot‐check methodology verified beam data consistency while reducing the need for extensive measurements. Measured TPR and OCR data were found in close agreement with the corresponding GBD model across all collimation systems and field sizes, with GI passing rates consistently exceeding acceptance criteria. Dosimetric verification of the hybrid GBD model using single‐beam irradiations yielded 100% GI passing rates at the 2%/1 mm acceptance criteria. Under the stricter 1%/1 mm criteria, 100% GI passing rates were maintained for all fields except the smaller apertures, where GI passing rates ≥ 97% were observed. Similarly, PSQA measurements revealed GI passing rates higher than 98%, 97% and 92% using GI acceptance criteria of 3%/1 mm, 2%/1 mm and 1%/1 mm, respectively.

The rationale underneath selective spot check validations provided in the CyberComm framework is based on the multi‐stage beam matching and characterization process. By first aligning the local linac source with the reference model through 60 mm fixed cone and primary beam tuning, the fundamental beam characteristics are securely matched. Accuracy across the clinical range of field sizes is further ensured by the mechanical precision of the fixed collimators and the specific calibration procedures for the variable apertures, which align field dimensions with GBD specifications. Furthermore, to mitigate risks associated with small‐field dosimetry — where deviations from reference data are most probable — fields with nominal diameters ≤ 10 mm (fixed and Iris) and ≤ 23.0 × 23.1 mm^2^ (MLC) are directly measured and merged into the model. Additionally, OF values for all field sizes and collimator types are explicitly measured and merged. The 100% GI passing rates observed during spot‐check validation suggest that the GBD model accurately represents the remaining intermediate field sizes, thereby obviating the need for comprehensive measurement of every aperture size once source matching and small‐field characterization are achieved.

GBD models in SRS and radiotherapy have been available for many years; for example, treatment planning in Gamma Knife (Elekta AB, Sweden) SRS relies on vendor‐integrated GBD models within the TPS.^36^ Moreover, O‐ring gantry systems (e.g., TomoTherapy and Halcyon) are supplied with predefined beam datasets, thereby limiting local measurements to a small set of verification tests.^11^, ^37^ Several studies have demonstrated that locally acquired beam data agree within 1% with manufacturer‐provided datasets^38^, ^39^ or with data from linacs of the same model.^40^, ^41^ Glide‐Hurst *et al* ^38^ reported excellent agreement among five Varian TrueBeam units across three institutions, with similar findings by Tanaka *et al*,^39^ while a recent multi‐institutional analysis showed that averaged data from 20 Varian C‐series linacs can serve as reference beam data for commissioning verification.^40^ However, GBD represents averaged beam characteristics and may not fully capture individual linac performance, particularly for small fields and advanced techniques. This limitation raises concerns regarding reduced sensitivity to machine‐specific discrepancies, potential inaccuracies in beam modelling, and the loss of detailed system understanding typically gained through comprehensive commissioning. Accordingly, GBD are generally recommended as a reference or starting point rather than a substitute for site‐specific beam data acquisition and validation, a position reflected in published Point/Counterpoint discussions in the peer‐reviewed literature.^42^, ^43^ Within the above context, the CyberComm framework represents a hybrid approach where the TPR and OCR profiles for the small apertures available with each collimator and the OF for all field sizes are explicitly measured ensuring consistency with the GBD model while preserving site‐specific dosimetric characteristics of the local linac.

Using the CyberComm framework a substantial reduction in commissioning time was achieved with all steps, including linac tuning, GBD spot‐checking, beam data commissioning for the small fields, OF measurements completed within 10 days. Compared to an estimated 25 days for conventional commissioning, this time saving corresponds to an efficiency gain of 60% without compromising dosimetric accuracy. Such time savings may have significant clinical implications, particularly in high‐throughput environments or during system installation and replacement. Moreover, besides reducing commissioning time and resource demands, the use of the CyberComm framework enhances inter‐institutional consistency while minimizing systematic errors associated with local measurements.

TG‐218 does not define specific gamma analysis parameters for SRS/SBRT cases, but advocates for tighter tolerances than those used for standard IMRT (3% DD, 2 mm DTA, 90% action threshold).^44^ TG‐135b recommends a 90% GI passing rate using 2%/2 mm criteria for the tumor, critical structures, and the high‐dose region down to the 50% isodose line.^34^ In this study, a stricter 1 mm DTA criterion was adopted, as tighter positional tolerances are more appropriate for SRS/SBRT quality assurance given the sub‐millimeter accuracy requirements of these treatments. Regarding dose calculation grid size, Medical Physics Practice Guideline 9.a recommends a maximum TPS calculation grid spacing of 1.25 mm for SRS, noting that 1 mm spacing may be necessary in certain cases.^45^ Xu *et al*,^35^ showed that doubling the dose grid size, affected the passing rates by less than 1%. In this work, a dose calculation grid of 0.55 × 0.55 × 1 mm^3^ was employed, satisfying these recommendations. The SRS MapCHECK detector dimensions of 0.48 × 0.48 × 0.03 mm^3^ are well within this grid resolution, minimizing potential spatial misregistration effects.^46^ In addition to the 3% DD criterion, stricter acceptance values of 2% and 1% were evaluated, consistent with similar studies in the literature.^35^, ^47^ Using a 3% DD criterion resulted in an average GI passing rate of 99.7%, which decreased to 99.3% and 96.1% when the DD was reduced to 2% and 1%, respectively. This progressive reduction in passing rates is likely attributable to the SRS MapCHECK measurement uncertainty, which has been reported to be of the order of 2%.^46^ Formal determination of GI passing rate tolerances and action levels for different GI acceptance criteria should be established through statistical process control procedures.^44^, ^48^ Nevertheless, and despite the small sample size, the findings of this study support the use of 2%/1 mm acceptance criteria with tolerance and action levels of 96% and 95%, respectively. If the stricter 1%/1 mm criteria are applied, the data suggest tolerance and action levels of 86% and 81%, respectively. However, guidance on tolerance and action levels for different GI acceptance criteria is beyond the scope of this work.

Despite the promising results of this work, certain limitations should be acknowledged. While the hybrid GBD approach reduced measurement workload, it inherently relies on the accuracy and stability of the reference dataset. Any systematic discrepancies in the GBD model could propagate into the commissioned system if not detected during spot‐check validation. In addition, MC commissioning is an iterative procedure in which the source model is determined based on the corresponding beam measurements (see Section 2.2).^19^ The source model is defined by three parameters: the source distribution, the photon fluence distribution, and the energy spectrum, which are iteratively adjusted until the MC‐calculated depth dose data and OCR profiles match the corresponding commissioned beam data for each secondary collimator system. This procedure is independent of the CyberComm framework and was therefore not included in this study. It should be noted, however, that since within the CyberComm framework the linac's beam characteristics are tuned to align with the reference dataset, appropriate starting values for the source model parameters should be used to converge to the optimal solution more efficiently. Finally, this study was conducted on a single CK system, and although results were consistent with vendor‐provided multi‐system reference data, further validation across multiple installations would strengthen the generalizability of the findings.

## 5 CONCLUSIONS

CyberComm‐assisted commissioning of the CK system enabled rapid, dosimetrically accurate, and standardized beam model generation. Beam data acquisition for all collimation systems was completed within 10 days, representing a substantial reduction in commissioning time relative to conventional workflows. The achieved dosimetric agreement in the single beam and PSQA tests confirmed that the CyberComm provides a reliable and efficient framework for CK beam commissioning while maintaining the high dosimetric accuracy required for stereotactic treatments. Although these results are based on a single center installation, they support the clinical feasibility of this workflow for CyberKnife commissioning.

## AUTHOR CONTRIBUTIONS

**E. Pantelis**: Conceptualization; methodology; data curation; formal‐analysis; figure preparation; writing manuscript. **A. Moutsatsos**: Data curation; writing manuscrip. **L. Sideri**: Data curation. **P. Archontakis**: Data curation. **A. Stergioula**: Review.

## FUNDING INFORMATION

The authors have nothing to report.

## CONFLICT OF INTEREST STATEMENT

The authors declare no conflicts of interest.

## ETHICS STATEMENT

No identifiable patient data were collected, and all procedures complied with the principles of the Declaration of Helsinki.

## Data Availability

The measured and analyzed data of the current study are available from the corresponding author upon reasonable request.
